# Retrospective Study of Leptospirosis in Malaysia

**DOI:** 10.1007/s10393-017-1234-0

**Published:** 2017-04-12

**Authors:** Bashiru Garba, Abdul Rani Bahaman, Siti Khairani-Bejo, Zunita Zakaria, Abdul Rahim Mutalib

**Affiliations:** 1 Department of Veterinary Pathology and Microbiology, Faculty of Veterinary Medicine, Universiti Putra Malaysia, 43400 Serdang, Selangor Malaysia; 2 Department of Veterinary Laboratory Services Unit, Faculty of Veterinary Medicine, Universiti Putra Malaysia, 43400 Serdang, Selangor Malaysia

**Keywords:** leptospirosis, Malaysia, retrospective study, emerging zoonoses

## Abstract

Leptospirosis is a bacterial disease transmitted to humans and animals by direct or indirect contact with urine or body fluids from infected animals especially rodents. Infection can be associated with wide clinical spectrum varying from asymptomatic to severe multi-organ syndrome with life-threatening consequences. We conducted a review of published studies on incidences, case reports, sero-epidemiological surveys from year 2000 to 2015 using different electronic data bases. Our study revealed that majority of the studies were conducted in Peninsular Malaysia and predominantly among high-risk human groups. Most of the studies on domestic animals were conducted in the 1980s; hence, the current status of leptospirosis among domestic animal population remains largely unknown. There tend to be a sharp rise in incidence rate among human population in the year 2014 which was attributed to flooding and heavy rainfall experienced as well as recreational activities. Several gaps in epidemiological knowledge were also disclosed.

## Introduction

Leptospirosis is a zoonotic infection caused by spirochaetes of the genus *Leptospira,* which according to genetically based classification has 19 species comprising of 13 pathogenic and 6 saprophytic *Leptospira* species (Adler and de la Peña Moctezuma 2010). The bacteria cause severe debilitating illness with fever, headache, joint pain, pulmonary haemorrhages and neurological complications (Faine et al. 1999). Human leptospirosis results from direct or indirect contact with contaminated urine, water or soil from infected animals. Leptospires are found throughout the world, but prevalence is higher in tropical regions with high rain fall (Haake and Levett 2015). Leptospirosis is a major public health concern, particularly in developing countries with limited economic resources. However, recent reports indicated its emergence as an important health risk in developed and developing countries including European countries, especially among individuals participating in water sport activities (Dupouey et al. 2014; Haake et al. 2002). Pathogenic *Leptospira species* which has 23 sero-groups and over 250 serovars are usually maintained in domestic and wild animal reservoirs, and this represents a persistent source of infection to livestock and humans cohabiting with them. Leptospirosis constitutes a significant financial constraint derived from human health cost and livestock production losses (Costa et al. 2015; WHO 2011). The global burden of leptospirosis is put at 0.10–975 cases per 100,000 populations and case fatality in the region of 6.85% depending on the prevalent serovars, healthcare services and economic status of the population (WHO 2015). A sound understanding of the epidemiology of leptospirosis in animal reservoir is a key factor in limiting the transmission to humans. Hence, controlling rodent populations which are the major source of human and domestic animal infection is critical.

The population of Malaysia as at July 2016 is 30,949,962 (Index Mundi, 39 2016), and based on statistical estimation of rat population (8 rats per person), the estimated rat population in Malaysia is 247, 599696 (Lim 2011). This alarming figure could possibly explain why the disease is endemic and continues to impact significantly on human health and well-being, livestock productivity and the national economy. Although the government has instituted disease control policies and implementation of prevention and control strategies, more efforts need to be done particularly in the control of reservoir animal. This report presents a review of available literature published from 2000 to 2015 relating to sero-epidemiological surveys, disease incidences in humans and animals and environmental-related studies. The main objective is to determine the current status of the disease in Malaysia from available literature and identify the relationship between environmental contamination, host animals and human infection.

### Approach

This study reviews relevant articles of both published and unpublished library materials that described the epidemiology, case reports, and outbreaks as well as cross-sectional studies of leptospirosis in Malaysia from 2000 to 2015. This is to enable us to identify discrepancies in the sero-prevalence of leptospires and its distribution in the environment. It also seeks to establish the existence of the relationship between environmental contamination and human and animal cases and other relevant epidemiological data. The methodology, search strategy and inclusion and exclusion criteria were based on laid out criteria derived by the authors that include relevance and significance of the studies. The principal data sources selected were PubMed, Google Scholar, Science Direct, Index Medicus for South East Asia Region (IMSEAR) and Leptospirosis Guidelines of the Malaysian Ministry of Health (MOH). The search language was restricted to English, and a combination of topic-related keywords using Boolean operators was used. Where necessary, parentheses, forward slash and asterisks were used to narrow the search results to only relevant articles according to our search criteria. In addition to the peer reviewed articles, newspaper reports, relevant theses and proceedings were also consulted in this review. Although the search protocol was restricted to articles published between years 2000–2015, an exception was made on articles reporting leptospirosis in domestic animals in Malaysia. This is because most of the published articles on prevalence of leptospirosis in domestic animals were in the 1980s, hence the need for modification. Thus, additional articles on animal leptospirosis were searched without any time frame.

### Literature Search

The literature search identified 186 relevant articles including peer reviewed, postgraduate thesis and guideline for control of leptospirosis in Malaysia, out of which 26 fulfilled the inclusion criteria for analysis. This review concentrates on national and regional epidemiological, incidence and outbreak reports including unpublished reports from Malaysian Ministry of Health. Although the combination of search words entered in each database was to retrieve relevant results, it may present bias (language, period of publication) and some important papers may have been overlooked. In addition, majority of the studies included in this review were cross-sectional studies reporting leptospirosis infection and associated risk factors at a specific point in time. Nevertheless, no study was excluded for qualitative reasons.

### Environmental Factors for the High Prevalence of Leptospiral Infection

Overseas, leptospirosis has been considered an occupational disease as a result of work-related activities that are considered as risk factors (Levett 2001). Other risk factors for acquiring leptospiral antibodies have been shown to be associated with exposure to house hold environment (Reis et al. 2008), infrastructural deficiencies related to open sewers, indiscriminate waste disposal and flooding. The environment in Malaysia is ideal for the survival and transmission of leptospirosis by virtue of its hot, humid and tropical climate with high rainfall. Rainfall tends to clean up rat holes bringing leptospires to the soil surface and water bodies. Majority of the reported incidences, outbreaks and serological surveys indicated direct linear relationship with environmental contamination. In other words, persistence of leptospirosis in a given locality is not entirely determined by high population of reservoir animals but the presence of fresh water and moist soil in suitable environment that supports the survival of pathogenic leptospires.

Khairani-Bejo and Oii (2004) conducted a study to determine survival of *Leptospira interrogans* serovar Hardjo in Malaysian environment. They analysed samples from six different sources of water (pond, drain, river, sea, rain and tap water), three different types of soils with varying degree of water retention capacity (sandy, loamy and clay) and urine samples exposed to different environmental conditions. Their investigation revealed a longer survival time of 11 days for *L. interrogans* serovar Hardjo in river water with pH of 6.7 to 7.3 placed under shade and the least survival was in sea water with acidic pH of 6.5 to 6.8. Although a longer survival time of 94 days had been reported by Hellstrom and Marshall (1978), survival of leptospires in water depends on the pH, salt concentration and viscosity (Trueba et al. 2004). Furthermore, Khairani-Bejo and Oii (2004) reported that leptospires survive in soil with high moisture content placed in shaded environment as compared to acidic soil with little moisture.

The second study comprised a cross-sectional sampling of two National Service Training Centers in Kelantan and Terengganu (Ridzlan et al. 2010). The investigation was to detect leptospires in the environment (soil and water) following earlier outbreak of leptospirosis in the camps. Of the total 145 water and soil samples collected from the two camps, 15 samples exhibited positive growths in modified EMJH medium (Johnson and Harris 1967) supplemented with fluoro-uracil, amounting to 10.34% out of which 3 (20%) were confirmed as pathogenic *Leptospira* based on 8-azaguanine test and PCR. Further analyses by microscopic agglutination test MAT revealed 2 (13.33%) belong to serovar Hebdomadis (Terengganu isolates). A higher detection of leptospires in Kelantan was attributed to higher rainfall experienced in the area especially during monsoon season. Many reports have also attributed increased incidence of leptospirosis to high rainfall (Levett 2001; Adler and de la Peña Moctezuma 2010; Bharti et al. 2003), and this may predispose animals and humans to increase cases of leptospirosis. In a related study, 151 water and soil samples were collected from recreational lakes and wet markets to detect leptospires. Thirty-five samples yielded positive growth, 8 of which were confirmed as pathogenic by PCR (Benacer et al. 2013b). The 23% prevalence reported in this study was slightly higher than the 20% reported by Ridzlan et al. (2010). The presence of more leptospires in effluents from wet market in this report could be attributed to indiscriminate waste disposal which usually attracts rodents and other domestic animals, thereby further contaminating the area. Although the prevalence in lakes was less than in drain waters according to this report, it still presents significant health risk particularly humans patronizing these facilities for recreation. Both studies presented evidence of high level of contamination of recreational parks and water bodies in close proximity with human dwellings. Differences in results are probably due to seasonal variations, study area location and nature of sample and methodology applied. The data also clearly demonstrated the risk of individuals being exposed to leptospires. Furthermore, the contamination level of two National Parks in Sarawak was investigated by Pui (Pui et al. 2015). Tanjung Datu National Park and Bako National Park are two of the most beautiful parks in Sarawak often visited by both foreign and local tourists. Out of the 110 environmental samples collected, 0.9% was found to be pathogenic and 5.5% intermediate. Although the prevalence is low compared to reports from other areas, it still disclosed the presence of the pathogen in the environment that would pose risk of exposure to the public and tourists visiting these parks (Pui et al. 2015). The degree of environmental contamination with leptospires depends on several factors including frequency, volume and concentration of leptospires in the urine, reservoir animal access to water sites, and water or soil type, temperature and movement of water. It can be seen from all the reports that leptospirosis is endemic in Malaysian environment and given the suitable prevailing conditions for survival, there is a need for intensive awareness campaign and monitoring to control the infection among human and animal populations.

#### Prevalence of *Leptospira* Infection Among Rodents in Malaysia

Rodents are considered the major reservoir of leptospires and the source of infection for humans and susceptible animals (Loan et al. 2015; Levett 2001). In Malaysia, majority of the serovars isolated are carried by rats (Bahaman and Ibrahim 1988). Khairani-Bejo and Oii (2004) reported 3.1% prevalence among rats caught in Serdang residential area. Both culture and MAT revealed negative results, while PCR detected 1 positive sample. This is a testament to the superior sensitivity of PCR over culture and MAT which are both laborious and time-consuming as reported by several authors (de Abreu Fonseca et al. 2006; Gravekamp et al. 1993; Noda et al. 2014). Recent study on isolation and identification of circulating leptospires among urban rats population in Kuala Lumpur (Benacer et al. 2013a) revealed 20 positive cultures out of 300 rats trapped, and polymerase chain reaction confirmed all 20 isolates to be pathogenic. Although the prevalence reported in this study is relatively low, the serovars identified by MAT (Bataviae and Javanica) are highly pathogenic. Hence, their presence in rats could pose risk to humans.

Mohamed Hassan et al. (2010) reported a prevalence of 17.3% and 18.3% leptospiral serovars by MAT in Kelantan and Terengganu, respectively, and the predominant serovar was Icterohaemorrhagiae. Unlike the previous study conducted in a market and residential areas, this study was conducted in National Service Training Centers in Kelantan and Terengganu states that have previously recorded outbreaks of leptospirosis. Because leptospirosis is associated with heavy rain and flooding, the high prevalence and diverse serovar distribution observed compared to earlier report (Benacer et al. 2013b) is not surprising. More so, Icterohaemorrhagiae is the predominant serovar isolated in rats similar to this study (Levett 2001). Additionally, 13% prevalence was also reported from wild rats in Johor (Latifah et al. 2012). In a related study conducted by the same authors in 2012 (Mohamed-Hassan et al. 2012), an overall prevalence of 12.3% was reported out of which 8.6% were pathogenic. Rats were caught from different locations (National Service Training Centres, Suburbs, oil palm estates and Royal Belum Rain Forest) and their kidneys harvested and cultured in semi-solid EMJH medium and analysed by PCR. Majority of the pathogenic leptospires were isolated from NSTC as previously reported (Mohamed Hassan et al. 2010). The lower prevalence recorded in this study compared with the earlier study may in part be as a result of larger sample size, location and prevailing environmental condition as at the time of sampling.

#### Leptospirosis in Domestic and Wild Animals

Domestic and wild animals are natural reservoirs and carriers of leptospires, which occasionally acts as maintenance and accidental hosts (Levett 2001). They usually develop chronic renal infection and continue to shed the organism in their urine for prolong period of time, contaminate the environment and expose other humans and animals to possible infection (Levett 2001). Leptospirosis in domestic and wild animals is reported to occur sporadically across a wide range of serovars (Bahaman et al. 1991). Majority of the pathogenic serovars circulating in Malaysia were isolated from wild rodents, and only six have been isolated in domestic animals (Bahaman et al. 1990). Cattle, buffalo and pigs have all been reported to have high prevalence 40.5, 31 and 16%, respectively (Bahaman et al. 1987). This report is similar to reports elsewhere where serovar Hardjo, Icterohaemorrhagiae, Hebdomadis, Canicola and Pomona were the predominant serovars isolated in domestic animals (Bharti et al. 2003; Romero-Vivas et al. 2013; Bahaman et al. 1987; Levett 2001). In another study, 14.4% was reported from urine cultures of 222 cattle, but serological survey yielded 45.5% prevalence among same number of cattle (Bahaman et al. 1988). This indicates that these animals are continually being exposed to leptospires in the environment and because the animals tend to serve as maintenance hosts, the effect is not really appreciated and as a result the animals may be suffering silently from the disease. The importance of domestic animals in the maintenance and transmission of leptospirosis cannot be overemphasized, hence the need for more efforts to determine the current status of leptospirosis among animal population in Malaysia. This is further highlighted by the high prevalence reported in some wild life in Sarawak (Siva et al. 2013). Although the sample size is arguably small (5 monkeys, 9 rats, 20 bats, 4 squirrels and 1 mongoose), the very high prevalence signifies the presence of the organism among wildlife and could be a potential source of infection to tourists. However, such outcomes could deter local and foreign tourists from visiting such centres which will inadvertently affect the economy and livelihood of the locals and the nation at large. A typical example is evidenced by the UK health authority travel advice to its citizens to avoid travel to all islands off the coast of eastern Sabah from Kudat to Tawau, including Lankayan, Mabul, Pom Pom, Kapalai, Litigan, Sipadan and Mataking due to the risk of Zika virus transmission (https://www.gov.uk/foreign-travel-advice/malaysia).

### Human Leptospirosis in Malaysia

The circle of transmission of leptospirosis involves a complex interaction between humans, animal reservoirs and the environment where they coexist (Lau et al. 2014). In rural areas, the disease is associated with farmers with increased risk during the warm and rainy season. In urban settlements on the other hand, leptospirosis is directly linked to poor hygiene, overcrowding and poverty (Lau et al. 2014; Thayaparan et al. 2013), while in recent times the disease has been reported to be associated with outdoor recreational activities and travelling especially in developed countries (Lau et al. 2010; Sejvar et al. 2003).

**Fig. 1:**
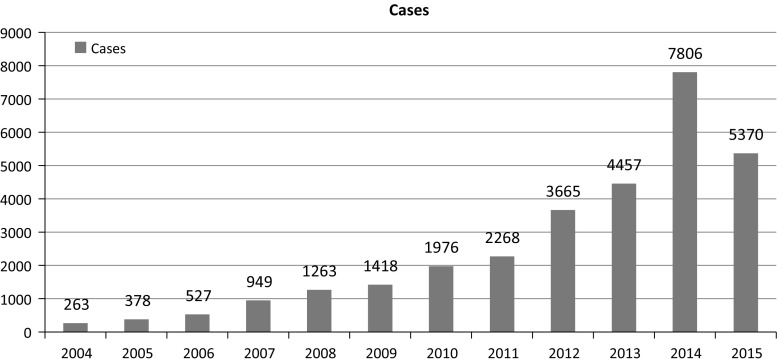
Number of leptospirosis cases in Malaysia from 2004 to 2015 (MOH 2015)

The majority of the articles have reported that leptospirosis is endemic in Malaysia (El Jalii and Bahaman 2004; Thayaparan et al. 2013; Lim et al. 2011). The high-risk group includes cleaners, abattoir workers, sewage workers, military personnel and recently individuals involved in recreational activities (Thayaparan et al. 2013; Sejvar et al. 2003). There has been a spike in the recorded cases of leptospirosis over the past ten years, and this has been attributed to better diagnostic techniques and awareness on the mode of transmission of the disease among Malaysians (Lim et al. 2011; Thayaparan et al. 2013). The ratio of male to female (ages between 20 and 60) infected with leptospirosis is 4:1 (Lim et al. 2011); this observation may be because males are mostly associated with the high-risk occupations earlier outlined. According to case fatality rate by states, Perak had the highest with 6.81% followed by Sarawak 6.42% and Perlis 6.25%. In September 2015, the sector head of zoonoses disease control division of the Ministry of health Malaysia (Dr. Zainudin Abdul Wahab) reported a progressive increase in recorded cases from 2004 to 2015 and highest number of deaths (92) was recorded in 2014 with a sharp decrease to 30 as at July 2015 (Proceeding of environmental health for local authorities 2015). The over 20% increase recorded from 2014 to 2015 may be as a result of the recent flood experienced in many parts of Malaysia as this has been reported to play a significant role in the spread of the disease in both human and animal populations (Fig. 1).

#### Outbreaks and Case Reports

**Fig. 2:**
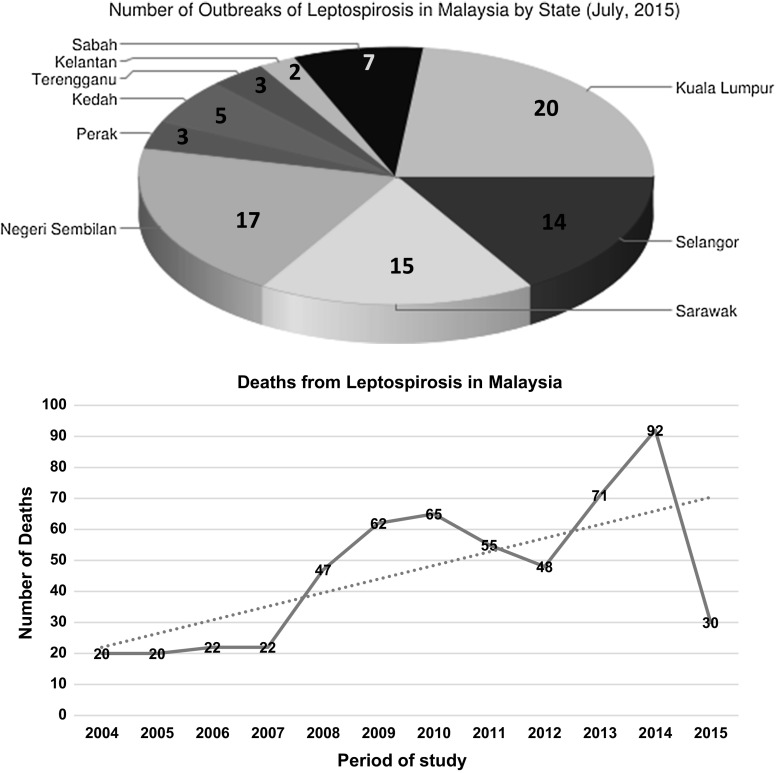
Number of death from leptospirosis during 2004 to July 2015 (MOH 2015)

The leptospirosis outbreak involving international participants in the Echo-challenge in the year 2000 in Borneo Island is recognized as the first international outbreak recorded (Sejvar et al. 2003). The outbreak involves 304 athletes from 26 countries. Although no death was recorded, 29 patients were hospitalized. This event serves to highlight the importance of recreation and water sports in the transmission of leptospirosis. In a related study, epidemiological investigation revealed leptospirosis as the cause of illness of 46 males admitted at the Beaufort Hospital Sabah after swimming in a creek near an oil palm plantation in Kampung, Kebatu, Beaufort (Koay et al. 2004). Unfortunately one death was recorded due to pulmonary haemorrhage, and 18 samples were positive by MAT test. The water body was suspected to have been contaminated by leptospiral organisms due to stagnation and flooding as a result of heavy rainfall in the area. Subsequently, locals were deterred from swimming in the creek as a precautionary measure. Similarly 252 in 2010, an outbreak of Melioidosis co-infection with leptospirosis involving 153 individuals who took part in a rescue mission to find the body of a young man who has allegedly drown in Lubuk Yu recreational forest was reported in Pahang. Among the ten confirmed Melioidosis cases, four were co-infected with leptospirosis (Sapian et al. 2012; Hin et al. 2012). Out of the eight people that died, seven were volunteer villagers and one professional rescuer. The overall case fatality was 70%, although this may have been aggravated by the fact that all the positive cases had diabetes mellitus (Sapian et al. 2012). Other significant finding in this study is that villagers had 100% case fatality rate compared to 33.3% for professional rescuers. As a result, non-professional rescue workers were discouraged to take part in the event of future occurrence (Fig. 2).

#### Sero-epidemiological Surveys

Most human infections are acquired via direct and indirect contact with infected urine or environment contaminated by urine; other important predisposing factors are occupational exposures and water sport activities (Narita et al. 2005; Haake and Levett 2015; Adler and de la Peña Moctezuma 2010; Haake et al. 2002). In May 2008, a cross-sectional serological survey was conducted among four high-risk occupations in Kota Bharu, Kelantan, namely garbage collectors, town cleaners, landscapers and lorry drivers. The overall prevalence was 24.7%, and garbage collectors and town cleaners had the highest prevalence 27.4% and 26.0%, respectively (Shafei and Sulong 2012). Furthermore, majority of the workers had poor knowledge of the disease and proximity to the river, rats infestation in houses and gardening activities are reported to be closely associated with increased cases of leptospirosis (Sulong et al. 2011; Aziah et al. 2012).

The annual incidence of leptospirosis is estimated at 0.1 to 10 in every 100, 000 people globally and could be higher in the event of flooding and heavy rainfall (Costa et al. 2015; WHO 2003; Pappas et al. 2008). This estimate may be erroneous due to frequent under reporting of the disease, lack of coordination and misdiagnosis of the disease (Pappas et al. 2008; Costa et al. 2015; Haake and Levett 2015). Six-month cross-sectional study was undertaken to determine leptospirosis among febrile patients admitted in 10 hospitals in north-eastern Malaysia (Rafizah et al. 2013a). Of the 111 samples obtained after screening of 999 samples using IgM ELISA, 8.4% were positive to MAT and higher prevalence was recorded for high-risk group individuals and those that were involved with recreational activities (Rafizah et al. 2013b). It was also discovered that leptospirosis is commonly misdiagnosed as reported by many authors (Kishimoto et al. 2004; Rafizah et al. 2012). Rafizah et al. (2013a) reported that only 31.0% of the leptospirosis confirmed cases were diagnosed as leptospirosis on discharge. However, 38.1, 14.3 and 7.1% were diagnosed as dengue fever, pneumonia and typhoid, respectively. This outlines the fact that overlapping of clinical manifestations with other febrile illnesses commonly results to erroneous neglect and misdiagnosis of leptospirosis which could result in increased mortality. Recently, Samsudin et al. (2015) conducted a sero-prevalence study among healthy municipal service workers to check for leptospiral antibodies using MAT. 34.8% of the 89 samples collected were positive. Garbage collectors had the highest prevalence of 41.5% followed by town cleaners 33.3% while samples from public workers and public health assistants were negative. Although the prevalence was high in garbage collectors and town cleaners, the result may not be unconnected with their job occupation as earlier outlined. Wearing of protective apparel has been shown to protect against brucellosis (Cook et al. 2016). Similarly, since leptospirosis is transmitted via cuts and skin abrasions, the use of protective equipment like gloves, boots and aprons is essential particularly among cleaners and garbage collectors whose job exposes them to the possibility of sustaining traumatic injury that may damage their skin making it easy for entry of viable leptospires inhabiting the environment. In a related study, 27% prevalence was reported among 100 healthy individuals in Ampang Jaya and the study also reveal lack of knowledge of leptospirosis among the respondents (Samsudin et al. 2015).

## Discussion

Leptospirosis is estimated to cause 1.03 million cases worldwide annually, and the estimated death is looking to exceed deaths caused by haemorrhagic fevers according to the Global Burden of Disease Study 2010 (Lozano et al. 2012; Costa et al. 2015). The disease is most common in resource poor tropical countries and is among the leading zoonotic causes of morbidity and mortality (Victoriano et al. 2009; Costa et al. 2015). Leptospirosis is a widely distributed bacterial infection in Southeast Asia, and despite the significant health and economic burden to human and livestock production, it is still a neglected and under reported disease (Sarkar et al. 2012; Laras et al. 2002; Thayaparan et al. 2013). The significance of this zoonotic disease is expanding, and the socioeconomic impacts are increasingly being experienced by many developing countries (King 2011). Available national data from the Malaysian Ministry of Health indicate a progressive increase in incidence and mortality from 2004 until 2014 (MOH 2015 unpublished). The high number of cases recorded in 2014 may be as a result of the flood that affects the north-eastern states of Malaysia with Kelantan, Perak and Selangor among the worst hit states.

Similarly, reports from our review tend to portray an increasing trend albeit with conflicting results. It is important to note that there seems to be a bias in the studies conducted in the period under review as majority of the reports are from north-eastern states of Malaysia, notably Selangor, Terengganu, Kelantan and to some extent Sabah and Sarawak from Borneo Island. Secondly, most of the samples were collected from high-risk group or from environment after flooding (Ridzlan et al. 2010; Samsudin et al. 2015). Flooding and occupation has been reported to be important factors that predisposes to leptospirosis (Levett 2001; Faine et al. 1999); hence, results from such studies may be misleading as it does not represent the true status of leptospirosis in the population. An exception is a study conducted among healthy municipal service workers in Selangor where 34.8% prevalence was reported (Samsudin et al. 2015). Again garbage collectors and town cleaners had the highest prevalence compared to public workers and public health assistants who had sero-negative results. While climatic changes and recent flooding may account for the increasing cases of leptospirosis, gazetting of leptospirosis as a notifiable disease by Malaysian Ministry of Health and improved diagnostic techniques and awareness may also be responsible for the high cases.

There was consistency in age group predisposition where majority fall under the category of 25–60 years (El Jalii and Bahaman 2004; MOH 2015 unpublished); this finding indicates possible high level of exposure in the community since this category forms the bulk of the work force particularly in the agricultural fields and other labour intensive jobs. There was also male predominance in the incidence data reported for the period under review (El Jalii and Bahaman 2004). This is slightly in contrast with 2010 annual Louisiana leptospirosis report which revealed higher cases between 25 and 34 years and continuous decline until 60 years. This may also reflect occupational exposure in male-dominated activities (WHO 2012). Maintenance of leptospires in domestic and wild animals is critical in the epidemiology of the disease. Different studies have identified rats, cattle, pigs and to a lesser extent sheep, goats and dogs as reservoirs of Icterohaemorrhagiae, Hardjo, Bratislava, Pomona and Canicola, respectively (Ellis 2015; Levett 2001; Hartskeerl and Terpstra 1996). In Malaysia, 38 different serovars have been bacteriologically identified (Bahaman and Ibrahim 1988; Blackmore et al. 1982; El Jalii and Bahaman 2004). Results from our review indicates consistency with reports elsewhere especially with regard to prevalence of Hardjo in cattle and buffalo, Pomona in sheep and goats, Icterohaemorrhagiae in rats and Bratislava in pigs and the prevalence range from 8.6 to 40.5% (Mohamed-Hassan et al. 2010, 2012; Benacer et al. 2013a; Bahaman 1991; Blackmore et al. 1982). Bahaman also observed infection of cattle and sheep with more than one serovar and was suggested to have resulted from co-grazing and intra-specie transmission of the agent within herd (Bahaman 1991). Although extensive research has been conducted in animal leptospirosis in Malaysia, efforts have shifted from domestic animal studies; hence, the current status remains unknown. This may explain why serovars not traditionally isolated in rats are being isolated (Benacer et al. 2013a) as these domestic animals continue to harbour and shed the pathogen in the soil and water.

Conclusively, from this review we have revealed a wealth of scientific evidence that supports the notion that leptospirosis is endemic in Malaysia and it is found in both human and animal populations as well as the environment. It also shows the diversity of serovar distribution in different localities, hence the need for further studies to determine their epidemiology and potential to cause disease in humans. The review also identified the lop-sidedness of research efforts in leptospirosis with more investigations (370) conducted in Peninsular Malaysia compared to the Borneo Island. Leptospirosis continues to be a major public health challenge globally. Ironically, there is still a lot not known about the disease and there are bountiful opportunities for quality leptospirosis research in Malaysia. To adequately control the menace of these important zoonoses, a one health approach bridging the medical, veterinary profession and the environmentalists is required.
